# YB-1 interplays with ERα to regulate the stemness and differentiation of ER-positive breast cancer stem cells

**DOI:** 10.7150/thno.41014

**Published:** 2020-02-19

**Authors:** Fan Yang, Siqi Chen, Shengnan He, Qin Huo, Ye Hu, Ni Xie

**Affiliations:** 1 Biobank, Shenzhen Second People's Hospital, First Affiliated Hospital of Shenzhen University, Shenzhen 518035, People's Republic of China; 2 Shenzhen institute of advanced technology, Chinese academy of sciences, Shenzhen 518035, People's Republic of China

**Keywords:** cancer stem cell, YB-1, ERα, stemness, differentiation

## Abstract

**Background:** Some stemness-associated transcription factors consistently play essential roles in the maintenance of pluripotency or induce the differentiation of cancer stem cells (CSCs). However, the regulatory mechanism of CSC stemness mediated by transcription factors has not been extensively explored. Here, we show that two transcription factors (YB-1 and ERα), which are simultaneously highly expressed in estrogen receptor (ER)-positive CSCs, interact with each other to regulate the stemness and differentiation of ER-positive CSCs.

**Methods:** The expression of YB-1 was examined in ER-positive CSCs and patient specimens. Western blot, real-time PCR, cell viability analysis, tumorsphere formation assay and subcutaneous tumorigenesis assays were used to study the stemness functions of YB-1 and ERα in CSCs. The relationship between YB-1 and ERα in cells was studied by promoter activity analysis, the electrophoretic mobility shift assay (EMSA) and the Co-IP assay. The mechanisms and functional significance of YB-1 in the sensitivity of CSCs to tamoxifen were further investigated with both in vitro and in vivo models.

**Results:** YB-1 was aberrantly upregulated in the cancerous tissue of ER-positive breast cancer patients and in CSCs. Knockdown of YB-1 in ER-positive CSCs significantly inhibited cell stemness and induced differentiation, and the expression of YB-1 could be regulated by estrogen signaling and ERα in ER-positive breast CSCs. The Co-IP results showed that YB-1 interacted directly with ERα specifically in ER-positive non-CSCs and that YB-1 induced ERα degradation by ubiquitination via direct interaction in differentiated cells. Cell differentiation induced by FBS could inhibit YB-1 phosphorylation and promote YB-1 protein transfer from the nucleus to the cytoplasm. Moreover, cell differentiation induced by targeting inhibited the expression of YB-1 in ER-positive CSCs, which increased the sensitivity of cells to tamoxifen in vitro and in vivo.

**Conclusion:** The ERα/YB-1 axis has an important role in the regulation of ER-positive breast cancer stemness. The dephosphorylation of YB-1 and the interaction between YB-1 and ERα may be the switch that initiates the differentiation of ER-positive CSCs. Targeting YB-1 to sensitize ER-positive CSCs to antiestrogen therapy might represent a new therapeutic strategy that warrants further exploration.

## Introduction

Breast cancer is a common type of malignant cancer and is the second-leading cause of cancer deaths in women 1. The growth of most breast cancers always depends on the effectiveness of estrogen and is controlled by estrogen receptor (ER)-induced signal transduction 2. These ERs receive signals from the estrogen molecule, leading to their dimerization and translocation to promote the growth of the cancerous cells 3. The functionality of the ER in breast cancer makes hormone therapy the major treatment for ER-positive breast cancer. Endocrine-based therapies, such as tamoxifen (TAM) 3 and aromatase inhibitors 4, have historically been used in clinical treatment to suppress ER function or inhibit estrogen biosynthesis. Although treatment with TAM has shown obvious benefits in most ER-positive breast carcinomas that are initially responsive to treatment, unfortunately, the repeated clinical use of endocrine-based therapies usually results in ER-positive breast cancer cell resistance to these treatments 5. Currently, TAM resistance is a serious challenge in the treatment of ER-positive breast cancer. The mechanism of increased resistance in breast cancer cells is unclear, and cancer stem cells (CSCs) are hypothesized to play an important role in this process 6.

CSCs, also known as “cancer-initiating cells”, are the driver of tumorigenesis and tumor development 7. During the occurrence and development of breast cancer, breast CSCs not only maintain their own number through self-renewal but also produce a large number of breast cancer cells with different phenotypes by rapidly proliferating and differentiating to promote the growth of breast tumors 8-10. Breast CSCs always maintain a dynamic balance between self-renewal and differentiation to maximize the growth needs of breast cancer. In breast cancer, CSCs have been prospectively isolated from primary tumors or cell lines based on their aldehyde dehydrogenase-positive (ALDH+) phenotype 11. As reported, ALDH+ CSCs with totipotency and differentiation features are thought to induce resistance to chemotherapy via their robust DNA damage repair proficiency, overexpression of ABC transporters or abnormal activation of numerous signaling pathways (e.g., the Notch, Hedgehog and Wnt pathways) 12-14. CSCs drive the different steps of the carcinogenesis process by self-renewing and differentiating, which promotes tumorigenesis and contributes to cellular heterogeneity 15-17. A recent report demonstrated that transcription factors regulate the self-renewal and differentiation of CSCs in various types of cancer 18. Like in early embryonic stem cells, many transcription factors, especially OCT4, NANOG, and SOX2, are overexpressed in CSCs 19-21. Overexpression of these genes (OCT4, NANOG, and SOX2) in human CSCs is associated with self-renewal, tumorigenicity and tumor metastasis 19-21. Several recent reports have also emphasized the effects of enhanced self-renewal and differentiation potential in ER-positive breast cancer when the ER signaling pathway is activated 22, 23. Estrogen treatment of ER-positive breast cancer cells was found to increase the tumorsphere formation capacity 22, 23. One proposed mechanism for this phenomenon is associated with the SOX2/NANOG/OCT4 self-renewal pathway; ERα was shown to directly bind to the promoter region of OCT4, potentially interfering with CSC self-renewal 22. These results suggest that activation of the ER receptor is related to stemness maintenance in ER-positive breast cancer. Interestingly, an increasing number of articles seem to emphasize that the ER signaling pathway is negatively correlated with the malignancy of breast cancer 3. Breast cancer cells with high ER expression are more sensitive to endocrine therapy drugs (e.g., TAM) than those with low ER expression, and the survival rate of ER-positive breast cancer patients is also better than that of ER-negative patients 2. There seems to be a contradiction between these results, and the molecular mechanisms remain poorly understood.

Y-box binding protein 1 (YB-1), which is encoded by YBX1, is an oncogenic transcription factor that binds both DNA and RNA to regulate DNA transcription, pre-mRNA splicing and DNA repair 24-27. YB-1 is also a major component of messenger ribonucleoprotein complexes, which stabilize mRNA and activate translation 28. The numerous functions of YB-1 indicate its important biological role in the occurrence and development of cancer. Indeed, the overexpression of YB-1 has been reported in various types of human cancers and is correlated with the expression of many cellular oncogenes 24-26. In addition to high expression, the function of YB-1 is also related to phosphorylation activation at the serine 102 position 27. Upon phosphorylation, YB-1 transfers from the cytoplasm to the nucleus, where it becomes an oncogenic transcription factor by inducing the expression of genes related to the unlimited growth, malignant transformation, cell invasion and multidrug resistance of cancer cells 28. Several additional phosphorylation sites on YB-1 have also been identified including Tyr281, Tyr162, Ser165 and Ser176, and each promot the expression of distinct target gene groups 24-26. Our previous studies found that YB-1 was significantly highly expressed in melanoma and breast CSCs and is involved in activation of the Notch and WNT signaling pathways, indicating that YB-1 is a factor that regulates CSC stemness 31, 32. Although important insights into the pluripotency function of YB-1 genes in CSCs have been studied, our findings have not impacted the knowledge regarding the regulatory mechanism of YB-1 expression in CSCs. Identifying factors that contribute to the overexpression of YB-1 in CSCs and can be targeted therapeutically thus has tremendous potential to significantly impact survival outcomes for breast cancer patients.

To address the above issue, we report two different regulatory functions of ERα and YB-1 in ER-positive breast CSCs and differentiated cells (non cancer stem cells, NCSCs) that maintain stemness and regulate the differentiation of ER-positive breast CSCs. This study provides new insight into CSC fate determination mechanisms, highlighting the unique dual regulatory roles of ERα and YB-1, and proposes a new targeted strategy for the treatment of ER-positive breast CSCs.

## Materials and methods

### Cell culture

The sorting of breast CSCs and NCSCs from the MCF-7 and ZR-75-1 breast cancer cell lines was conducted using an ALDEFLUOR^TM^ kit (Cyagen Biosciences Inc., USA) according to our previous report 31, 32. Breast CSCs were cultured in DMEM/F-12 (Invitrogen, USA) supplemented with 20 ng/mL epidermal growth factor (Beyotime Biotechnology, Jiangsu, China), 10 ng/mL basic fibroblast growth factor (Beyotime, China), 5 μg/mL insulin (Beyotime, China) and 2% B-27 (Sigma, USA) at 37 °C in a humidified atmosphere with 5% CO_2_. MCF-7 breast cancer cells and NCSCs were cultured in MEM supplemented with 10% fetal bovine serum (FBS) (GIBCO, USA), 2 nM glutamine (Thermo Fisher Scientific, USA) and 5 μg/mL insulin (Beyotime, China) at 37 °C in a humidified atmosphere with 5% CO_2_. ZR-75-1 breast cancer cells and NCSCs were cultured in DMEM supplemented with 10% fetal bovine serum (FBS) (GIBCO, USA) at 37 °C in a humidified atmosphere with 5% CO_2_.

### Immunohistochemistry analysis

To detect YB-1 protein expression in human breast cancer and adjacent tissues by immunohistochemical staining, 5-μm-thick sections were placed on precoated slides with 3-(triethoxysilyl)propylamine (Merck, Darmstadt, Germany). The Slides were soaked in xylol for 1 h and washed in a series of alcohols with decreasing concentrations. After tissue deparaffinization, antigen retrieval was performed on the sections in a microwave for 5 min in TEC buffer (0.05 M Tris-HCl, 0.05 M ethylenediaminetetraacetic acid, 0.02 M Na-citrate (pH 7.8)), followed by blocking in peroxidase. After incubation with a primary antibody against YB-1 for 12 h, the slides were incubated with a biotinylated rabbit anti-mouse antibody (Vector, Grünberg, Germany) for 30 min. Subsequently, the slides were stained with diaminobenzidine (DAB) (Sigma, USA) for 10 min to label YB-1 proteins and then counterstained with hematoxylin to label nuclei. Cancerous and paracancerous tissue from patients with ER-positive breast cancer were obtained from Shenzhen Second People's Hospital, Shenzhen University. All human breast cancer sample acquisitions were approved by the Committee on Ethics of Shenzhen Second People's Hospital, Shenzhen University. Written informed consent was obtained from all the participants.

### Kaplan-Meier plotter database analysis

The correlation of the YB-1 transcription level and overall survival in ER-positive breast cancer was analyzed by the Kaplan-Meier plotter database (http://www.kmplot.com/). A probe (ID: 208627_s_at) containing 2061 ER-positive breast cancer patients was used (YB-1-low: n=1030; YB-1-high: n=1031). The hazard ratios (HRs) with 95% confidence intervals and logrank p value were also computed.

### Gene knockdown in cells

To transiently knock down the expression of YB-1 and ERα in CSCs, an RNAi assay was conducted using YB-1-specific siRNA (Supplementary Table 1) and ERα-specific siRNA (Supplementary Table 1). Cells (1×10^5^) were transfected with 50 nM siRNA using Lipofectamine 2000 (Invitrogen, USA). All siRNAs were synthesized by Shanghai GenePharma Co., Ltd. (Shanghai, China). At different times after transfection, cells were harvested for later use.

To establish the stable YB-1-silenced MCF-7 CSC line, two short hairpin RNA (shRNA) (Supplementary Table 1) plasmid constructs cloned into the retroviral pGFP-V-RS vector were used. After plasmid transfection, cells were cultured by replacing the medium every three days with complete medium containing 1 µg/mL or 0.5 µg/mL puromycin, alternating each week, for one month, during which stable clones were selected and passaged for later experiments. The expression of YB-1 was measured in the selected clones using real-time PCR.

### Expression of cellular proteins

To express exogenous proteins (YB-1 and ERα) in cells, the protein-coding genes were cloned into the pcDNA3.1(+) plasmid (Invitrogen, USA). Then, cells were transfected with 150 nM recombinant plasmid using Lipofectamine 2000 (Invitrogen, USA). The expression levels of the exogenous proteins were assayed by western blotting.

### Western blot analysis

The protein levels of some genes in cells were measured by western blotting. Proteins were separated using 12% SDS-PAGE and were then transferred to a polyvinylidene fluoride (PVDF) membrane. The membrane was blocked with triethanolamine-buffered saline solution (TBS) containing 5% skim milk. Subsequently, the membrane was incubated with antibody overnight and was then incubated with alkaline phosphatase-conjugated secondary antibody (Roche, Switzerland) for 2 h at room temperature. After the membrane was rinsed, immunoreactions were detected with BCIP/NBT substrate (Sangon Biotech, Shanghai, China).

### Quantification of mRNA by real-time PCR

Total RNA was extracted using an RNA isolation kit (Ambion). The reverse transcription reaction was conducted using a PrimeScript™ RT Reagent Kit (Takara, Japan). Quantitative real-time PCR was performed with 2× TaqMan Premix Ex Taq (Takara, Japan) to detect genes. Real-time PCR was performed according to our previous report 24. The sequences of the primer pairs are shown in Supplementary Table 1.

### Cell viability analysis

To assess the viability of cancer cells, the change in the cell number was determined. A total of 1 × 10^4^ cells were seeded in a 6-well plate in 1 ml of culture medium. After culture for different times at 37 °C in a humidified incubator containing 5% CO_2_, cells were counted with a Scepter™ 2.0 Cell Counter. All experiments were repeated three times.

### Tumorsphere formation assay

A tumorsphere formation assay was conducted under nonadherent and serum-free conditions to examine CSC stemness. Cells were suspended in CSC culture medium, and a single cell was then plated in an ultralow attachment 96-well plate and cultured for 7 days with examination under a light microscope. After the primary mammospheres were established, the tumorspheres were blown away to form single cells and continued to be cultured until the secondary and tertiary mammospheres were established. For the differentiation experiment, 1% FBS (GIBCO, USA) was added to CSC culture medium to induce cell differentiation.

### Promoter activity analysis

The promoter sequence of the YB-1 gene was cloned into the pGL3 firefly luciferase reporter vector (Promega, USA) with sequence-specific primers (Supplementary Table 1). Then, the recombinant pGL3 vector was cotransfected into MCF-7 breast cancer stem cells with the pRL-TK Renilla luciferase reporter vector (Promega, USA). The firefly luciferase activity of pGL3 and Renilla luciferase activity of pRL-TK were measured in accordance with the dual luciferase reporter assay protocol recommended by Promega. Cells were washed with PBS and lysed in passive lysis buffer (Promega, USA) for 30 min. Subsequently, the cell lysate was subjected to detection of firefly luciferase activity (M1), followed by detection of Renilla luciferase activity (M2). The promoter activity was calculated according to the following formula: Promoter Activity=M1/M2.

We also examined the effect of ERα expression on YB-1 promoter activity. The ERα protein-coding gene sequence was cloned into the pcDNA3.1 plasmid (Invitrogen, USA). Then, cells were transfected with 150 nM recombinant plasmid using Lipofectamine 2000 (Invitrogen, USA).

### Screening for proteins bound to the YB-1 promoter

To screen for proteins bound to the YB-1 promoter, a biotin-streptavidin pulldown assay was conducted. The YB-1 promoter was labeled with biotin (Thermo Scientific, USA). MCF-7 breast CSCs were harvested and washed with cold PBS three times. Then, cells were lysed in lysis buffer (5 mM piperazine-1,4-bisethanesulfonic acid, 85 mM KCl, and 0.5% Nonidet P-40) containing 2 mM phenylmethanesulfonyl fluoride (PMSF) and were then incubated for 30 min on ice. The cell lysate was incubated with the biotin-labeled YB-1 promoter and streptavidin-coupled agarose beads (Thermo Scientific, USA) at 4 °C overnight. After three washes with PBS, the mixture was resuspended in RIPA lysis buffer (Beyotime Biotechnology, China) and heated at 100 °C for 5 min. The mixture was centrifuged for 5 min at 15,000×g, and the supernatant was analyzed by western blotting.

### Electrophoretic mobility shift assay (EMSA)

EMSA was performed to evaluate the binding of ERα protein with the YB-1 promoter. ERα protein was purified and incubated with the YB-1 promoter at different concentrations. After incubation in the reaction buffer (0.1 M KCl, 1 mM dithiothreitol, 1 mM MgCl_2_, 10 mM HEPES (pH 7.6)) at 37 ℃ for 30 min, the mixture was separated by 1% agarose gel electrophoresis at 100 V for 25 min and was then stained with ethidium bromide to examine DNA.

### Coimmunoprecipitation (Co-IP) assay

Cells were harvested by centrifugation at 300×g for 10 min and lysed with ice-cold cell lysis buffer (Beyotime Biotechnology, China). An antibody specific for the target protein was incubated with Protein G-agarose beads (Invitrogen, USA) for 3 h at 4℃, and the lysate was then added to the mixture. The mixture was incubated at 4℃ for 12 h. After being washed three times with ice-cold PBS, the Co-IP product was collected, and proteins were analyzed by western blotting.

### Construction of the YB-1/Tet-On plasmid

The YB-1 protein-coding gene sequence was obtained from the pcDNA3.1-YB-1 plasmid. TRE-Tight, rtTA, and YB-1-3×NLS (AGATCCAAAAAAGAAGAGAAAGGTAGATCCAAAAAAGAAGAGAAAGGTAGATCCAAAAAAGAAGAGAAAGGTAGATACGGCC)-3×FLAG sequences were inserted into the BamHI-EcoRV sites of the pEB-multi vector. After the plasmid was constructed, 0.1 μg/mL doxycycline (Dox) was added, and the expression level of Flag-YB-1 in cells was examined by western blotting.

### The ubiquitination level of ERα

Cells were lysed in lysis buffer (5 mM piperazine-1,4-bisethanesulfonic acid, 85 mM KCl, and 0.5% Nonidet P-40) and incubated for 30 min on ice. Anti-ERα monoclonal antibody (Cell Signaling Technology, USA) was incubated with Protein G-agarose beads (Invitrogen, USA) for 3 h at 4℃, and the lysate was then added to the mixture. After incubation at 4 ℃ for 12 h, the mixture was washed with ice-cold PBS three times. Finally, the product was analyzed by western blotting with an anti-ubiquitin antibody (Cell Signaling Technology, USA).

### Cell cycle analysis

Cell cycle analysis was conducted via flow cytometry. After cell samples were fixed in ice-cold ethanol for 4 h, cells were incubated with DNase-free RNase A (20 μg/mL) for 30 min. Then, cells were centrifuged at 500 × g for 5 min and stained with propidium iodide (PI) (50 μg/mL). The fluorescence intensity of 1 × 10^4^ cells was measured with a flow cytometer at an excitation wavelength of 488 nm.

### Analysis of caspase 3/7 activity

A Caspase-Glo 3/7 assay (Promega, USA) was used to evaluate the activity of caspase 3/7 according to the manufacturer's protocol described in our previous study 31, 32.

### Tumorigenicity in nude mice

Nonobese diabetic/severe combined immunodeficiency (NOD/SCID) female mice weighing ~25 g and aged ~5 weeks were used for the in vivo experiments. YB-1-knockdown MCF-7 breast cancer stem cells and wild-type MCF-7 breast cancer stem cells were collected at 5 × 10^5^ cells/mL in physiological saline. Matrigel (Becton, Dickinson and Company, USA) was added to the cell suspension at a ratio of 1:2. Subsequently, 200 μL of the cell suspension was subcutaneously injected into mice to induce tumor growth. Twenty-five days later, tamoxifen was administered via intragastric gavage at a dosage of 3 mg/kg/day for 30 days. The tumor sizes were measured with a caliper every 5 days, and the tumor volume was calculated as (length×width×width)/2. Sixty days later, the mice were sacrificed by cervical dislocation, and the solid tumors were collected by detachment using surgical scissors. The tumor sizes and weights were examined. Animal experiments were approved by the Animal Ethics Committee of Shenzhen University, China. All methods were carried out in accordance with the approved guidelines.

### Statistical analysis

All numerical data are presented as the means ± standard deviations. The data were processed using one-way analysis of variance (ANOVA), and Student's t-test was employed to assess significant differences. All assays were performed in biological triplicate.

## Results

### YB-1 regulates the stemness of ER-positive breast CSCs

**Figure 1:**
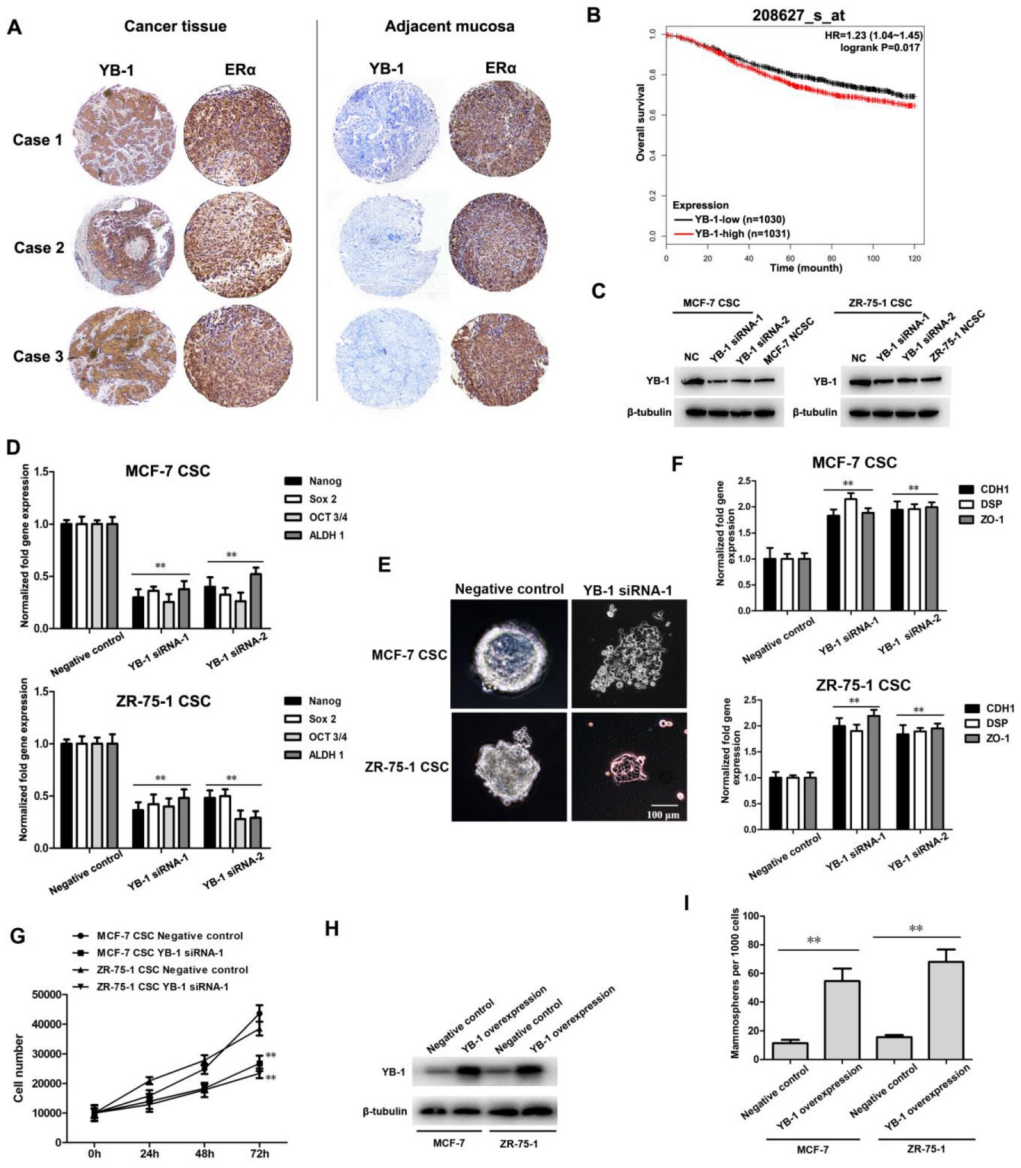
** YB-1 regulates the stemness of ER-positive breast CSCs.** (A) Immunohistochemical analysis of the YB-1 protein in ER-positive breast cancer tissue and adjacent mucosal tissues. The YB-1 and ERα proteins were detected with a YB-1-specific antibody and ERα-specific antibody (brown), respectively. Nuclei were stained with hematoxylin (blue). (B) The Kaplan-Meier database was used to predict the relationship between the mRNA level of YB-1 and overall survival of ER-positive breast cancer patients. A probe (ID: 208627_s_at) containing 2061 ER-positive breast cancer patients was used (YB-1-low: n=1030; YB-1-high: n=1031). HR=1.23, log rank P=0.017. (C) The expression of YB-1 in ER-positive breast CSCs and NCSCs. Two YB-1 siRNAs were used to knock down YB-1 expression in CSCs. The YB-1 protein was detected by western blotting. β-Tubulin was used as the control. NC indicates the negative control. (D) Effects of YB-1 knockdown on the expression of stemness-related genes in CSCs. Thirty-six hours after transfection with YB-1 siRNA, quantitative real-time PCR was used to evaluate the expression levels of stemness-related genes (**, *p*<0.01). (E) Influence of YB-1 knockdown on the tumorsphere formation capacity of ER-positive breast CSCs. Seventy-two hours after transfection with YB-1 siRNA, cells were examined under a light microscope. Scale bar, 100 μm. (F) Impact of YB-1 knockdown on the expression of differentiation-related genes in ER-positive breast CSCs. Thirty-six hours after transfection with YB-1 siRNA, the expression levels of differentiation-related genes in cells were examined by quantitative real-time PCR (**, *p*<0.01). (G) The examination of cell number. ER-positive breast CSCs were seeded in a 6-well plate at 1×10^4^ cells/well. At different time points after transfection with YB-1 siRNA, the cells were counted. The experiments were carried out in triplicate (**, *p*<0.01). (H) The expression of YB-1 in MCF-7 and ZR-75-1 cells. Thirty-six hours after transfection with the pcDNA-YB-1 plasmid, the YB-1 protein was detected via western blotting. β-Tubulin was used as the control. (I) Influence of YB-1 overexpression on the tumorsphere formation capacity of MCF-7 and ZR-75-1 cells. The tumorsphere formation rate per 1000 cells was calculated (**, *p*<0.01).

To determine whether YB-1 plays a role in ER-positive breast cancer, the expression patterns of the YB-1 protein in ER-positive breast cancer patients were examined by immunohistochemistry analysis. The data demonstrated that YB-1 showed a high expression level in ER-positive breast cancer tissue compared with adjacent mucosal tissue (Figure 1A). Kaplan-Meier survival analysis showed that ER-positive breast cancer patients with high YB-1 expression had significantly poorer short-term (0-10 years) overall survival rates than patients with low YB-1-low expression (Figure 1B). These results indicated that high YB-1 levels are correlated with the development and poor clinical outcome of ER-positive breast cancer.

Human ER-positive breast CSCs were sorted from MCF-7 and ZR-75-1 cells using ALDH1 (a stem cell marker) and further confirmed by assessment of the tumorsphere formation ability (Supplementary Figure S1A) and in vivo tumorigenicity (Supplementary Figure S1B). To elucidate the role of YB-1 in ER-positive breast CSCs, the expression level of YB-1 in breast CSCs and NCSCs was investigated. Western blot data showed that YB-1 was significantly upregulated in ER-positive breast CSCs compared with NCSCs (Figure 1C).

To determine the effects of YB-1 on the stemness of ER-positive breast CSCs, the expression levels of stemness-related genes and the tumorsphere formation capacity were evaluated in YB-1 knockdown and YB-1 negative control ER-positive breast CSCs. Western blot analysis showed that YB-1 expression was decreased in YB-1 knockdown breast CSCs (Figure 1C). The expression levels of stemness-related genes, including Oct-3/4, Nanog, ALDH1 and Sox2, were significantly downregulated in YB-1 knockdown CSCs compared with negative control cells (Figure 1D). Furthermore, YB-1 knockdown CSCs lost their tumorsphere formation capacity and showed adherent growth with differentiation morphology (Figure 1E). To investigate whether downregulation of YB-1 in ER-positive breast CSCs actually leads to cell differentiation, the expression levels of differentiation-related genes (CDH1, DSP and ZO-1) in breast CSCs were examined 33-35. YB-1 knockdown significantly promoted the expression of these differentiation-related genes (Figure 1F), indicating that YB-1 knockdown induces loss of stemness and promotes the differentiation of ER-positive breast CSCs. To explore the role of YB-1 in CSCs, the proliferation of YB-1 knockdown and YB-1 negative control breast CSCs was evaluated. The cell viability assays revealed that the viability of YB-1 knockdown CSCs was significantly decreased compared with that of YB-1 negative control CSCs (Figure 1G). To further evaluate the effects of YB-1 on the stemness status of ER-positive breast cancer cells, exogenous YB-1 protein was overexpressed in wild-type MCF-7 and ZR-75-1 breast cancer cells. Western blot analysis showed that YB-1 expression was increased in YB-1-overexpressing MCF-7 and ZR-75-1 breast cancer cells compared with negative control cells (Figure 1H). As shown by overexpressing YB-1 in normal breast cancer cells, the tumorsphere formation rate per 1000 cells was significantly increased compared with that in negative control cancer cells under serum-free conditions (Figure 1I). These data further indicate that YB-1 can enhance the stemness of ER-positive breast cancer cells.

Taken together, these findings indicate that YB-1 is required for stemness and that YB-1 knockdown promotes the differentiation of ER-positive breast CSCs.

### ERα and estrogen signaling induce YB-1 expression

**Figure 2:**
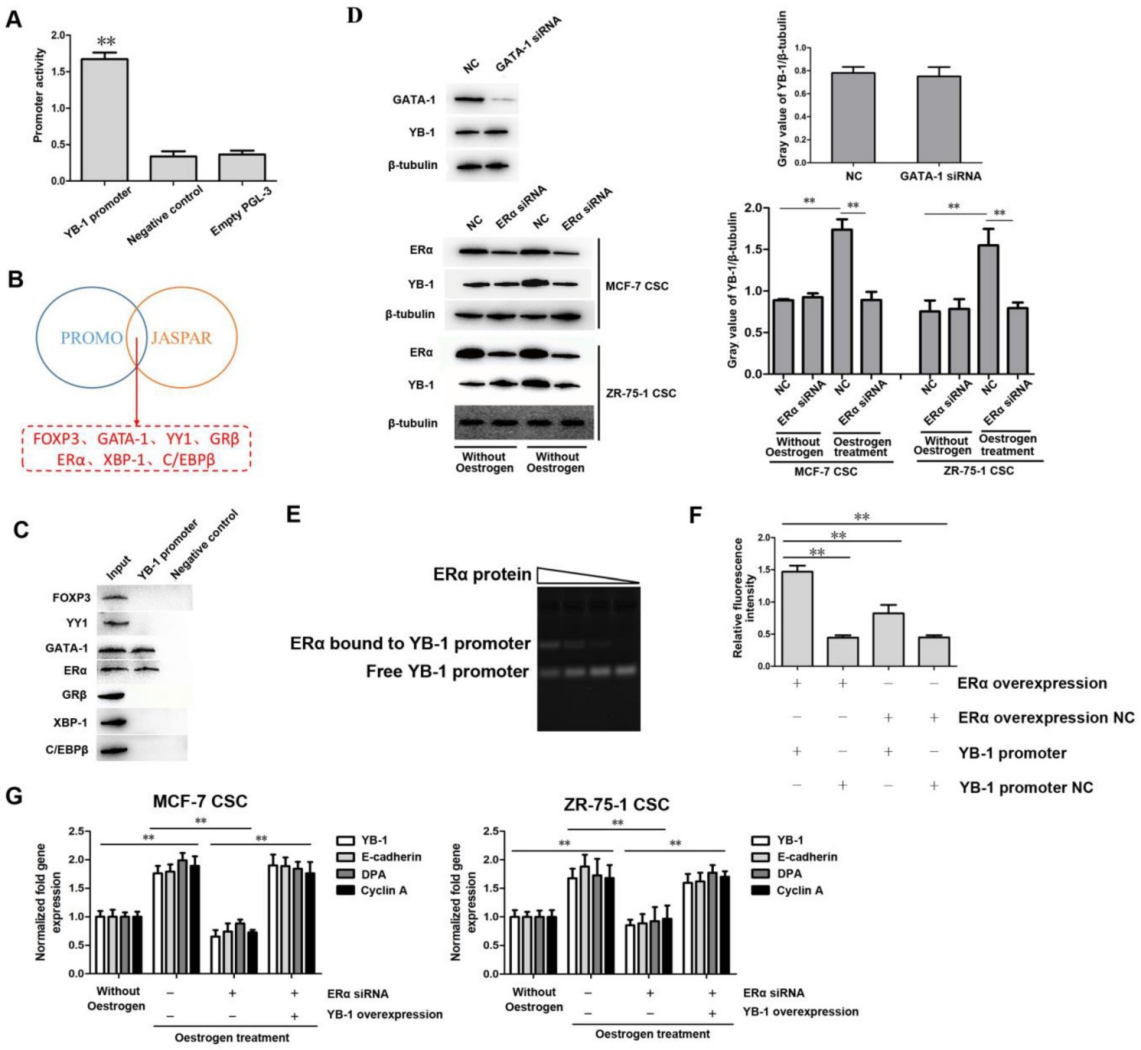
** ERα and estrogen signaling induce YB-1 expression.** (A) Assessment of YB-1 promoter activity. A plasmid containing the YB-1 promoter and the control pRL-TK plasmid were cotransfected into MCF-7 CSCs. Thirty-six hours after transfection, firefly luciferase and Renilla luciferase activity was analyzed to evaluate promoter activity (**, *p*<0.01). (B) Prediction of the potential transcription factor binding sites in the YB-1 promoter by the PROMO and JASPAR databases. (C) DNA-protein pulldown experiment to reveal the potential TFs for YB-1. After the YB-1 promoter was labeled with biotin and incubated with whole-cell lysate, proteins were detected by western blotting. (D) Influence of GATA-1 and ERα knockdown on the expression level of YB-1. Thirty-six hours after transfection with GATA-1 and ERα siRNA, western blotting (left) was used to evaluate the expression level of YB-1, and β-Tubulin was used as the control. The gray value of YB-1/β-tubulin (right) was calculated by Image J software (**, p<0.01). Oestrogen (10 nM) was used to activate ERα signaling. NC means negative control. (E) Direct interaction between the ERα protein and the YB-1 promoter. After incubation of the YB-1 promoter with the ERα protein, the mixture was separated on a 1% agarose gel and stained with ethidium bromide to visualize the DNA. The wedges indicate the concentration gradient of the ERα protein used. (F) The direct interaction between ERα and the YB-1 promoter. MCF-7 CSCs were cotransfected with the pcDNA-ERα plasmid and a plasmid containing a luciferase reporter fused with the YB-1 promoter. Thirty-six hours after transfection, firefly and Renilla luciferase activity was analyzed. As controls, pcDNA-ERα negative control and YB-1 promoter negative control were included in the cotransfections (**, *p*<0.01). (G) Impact of ERα signaling on the expression of YB-1 target genes in ER-positive breast CSCs. Thirty-six hours after transfection with pcDNA-YB-1 and/or ERα siRNA, the expression levels of YB-1 target genes in cells were measured by quantitative real-time PCR. Estradiol (10 nM) was used to activate ERα signaling (**, *p*<0.01).

Analysis of the promoter region of the human YB-1 gene in a previous study showed that the region between positions -119 and +127 may act as a core promoter sequence for transcription 36. To further confirm the transcriptional activity of this region, we cloned the sequence between positions -119 and +127 into the pGL3 firefly luciferase reporter vector, and this region showed strong promoter activity compared with the control (Figure 2A). To explore the mechanism underlying the high expression of YB-1 in ER-positive breast CSCs, the upstream transcription factor binding sites in the YB-1 promoter were predicted using the PROMO (http://alggen.lsi.upc.es) and JASPAR (http://jaspar.genereg.net) databases. The results showed that 7 proteins (FOXP3, GATA-1, YY1, GRβ, ERα, XBP-1, and C/EBPβ) were potential transcription factors for YB-1 (Figure 2B). We further performed a DNA-protein pulldown experiment to reveal the transcription factors upstream of YB-1. The western blot results showed that only two of the seven transcription factors (ERα and GATA-1) could bind to the YB-1 promoter (Figure 2C). To explore the effects of ERα and GATA-1 on the expression of YB-1 in ER-positive breast CSCs, ERα and GATA-1 siRNA were transfected into cells. The western blot results showed no significant effect on the expression of YB-1 after GATA-1 knockdown in MCF-7 CSCs (Figure 2D). Conversely, ERα knockdown led to a significant decrease in YB-1 expression in MCF-7 and ZR-75-1 CSCs in the presence of oestrogen (Figure 2D). Furthermore, the expression level of YB-1 was significantly increased by oestrogen treatment in MCF-7 and ZR-75-1 CSCs (Figure 2D). These results indicate that the expression and activation of ERα can induce the expression of YB-1 in ER-positive CSCs.

To explore the mechanism underlying the regulation of YB-1 expression by ERα in ER-positive CSCs, an electrophoretic mobility shift assay, in which the YB-1 promoter was incubated with the ERα protein was conducted and revealed that the ERα protein directly bound to the YB-1 promoter (Figure 2E). To explore the interaction between ERα and the YB-1 promoter, dual luciferase reporter assays were conducted in MCF-7 CSCs. The luciferase activity in the cells transfected with the PGL3-YB-1 promoter and pcDNA-ERα plasmid was significantly increased compared with that in control cells (Figure 2F), showing that ERα directly interacted with the YB-1 promoter. To evaluate the influence of the ERα protein on the expression of YB-1 downstream genes, we analyzed three of the YB-1 target genes (E-cadherin, DPA, and Cyclin A) 29, 30, 37. The quantitative real-time PCR results showed that the mRNA levels of these genes (E-cadherin, DPA, and Cyclin A) were significantly upregulated under oestrogen treatment and that knockdown of ERα inhibited the expression of these genes in CSCs (Figure 2G). However, when YB-1 expression was rescued, these genes (E-cadherin, DPA, and Cyclin A) still showed high expression levels even when ERα was knocked down (Figure 2G). These results indicate that ERα is the transcription factor for YB-1 in ER-positive breast CSCs.

### Role of the ERα/YB-1 axis in the regulation of breast cancer stemness

**Figure 3:**
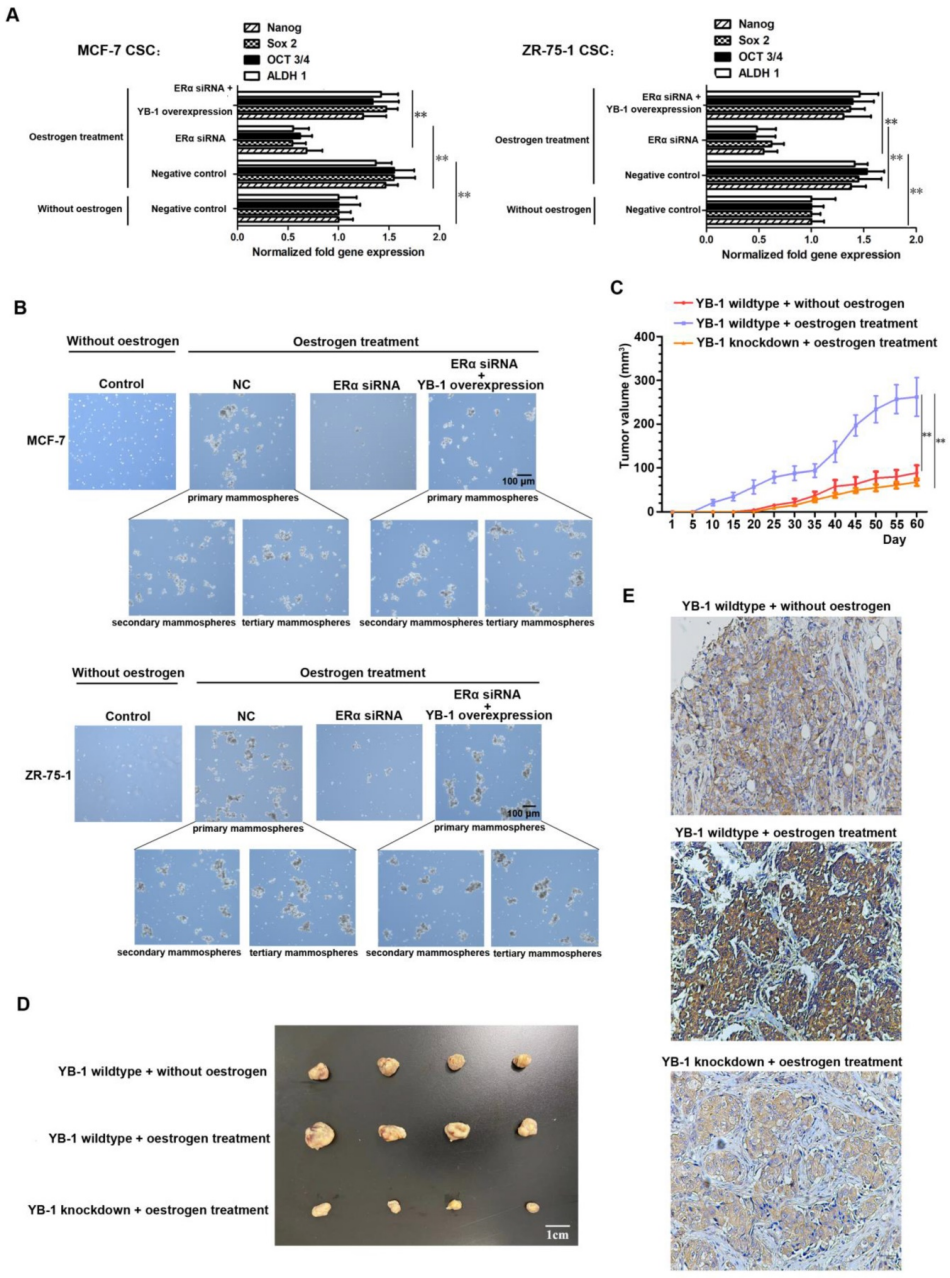
** Role of the ERα/YB-1 axis in the regulation of breast cancer stemness.** (A) Impact of ERα signaling on the expression of stemness-related genes in ER-positive breast CSCs. Thirty-six hours after transfection with pcDNA-YB-1 and/or ERα siRNA, the expression levels of stemness-related genes in cells were measured by quantitative real-time PCR. Oestrogen (10 nM) was used to activate ERα signaling (**, *p*<0.01). (B) Influence of ERα/YB-1 axis on the tumorsphere formation capacity of ER-positive breast CSCs. Seventy-two hours after transfection with pcDNA-YB-1 and/or ERα siRNA, cells were examined under a light microscope. Tertiary mammospheres were developed to demonstrate the self-renewal capacity and stemness of the cells. Oestrogen (10 nM) was used to activate ERα signaling. Scale bar, 100 μm. NC means negative control. (C and D) Influence of the ERα/YB-1 axis on the tumorigenic capacity of ER-positive breast CSCs. Estrogen sustained release tablets were implanted subcutaneously to activate ERα signaling. The tumor volumes in the mice were measured every 5 days (C) (**, *p*<0.01). Sixty days later, the mice were sacrificed. A solid tumor was collected from each mouse (D). (E) Immunohistochemical analysis of the YB-1 protein in tumors. The YB-1 protein was detected with a YB-1-specific antibody (brown). Nuclei were stained with hematoxylin (blue).

We further explored whether ERα can affect the stemness of CSCs by regulating the expression of YB-1. The quantitative real-time PCR results revealed low expression levels of stemness-related genes (Oct-3/4, Nanog, ALDH1 and Sox2) in ERα knockdown CSCs in the presence of oestrogen (Figure 3A). However, when YB-1 expression was rescued, all of the stemness-related genes were simultaneously highly expressed in MCF-7 and ZR-75-1 CSCs (Figure 3A). We also examined the influence of the ERα protein and ER signaling on the tumorsphere formation capacity of MCF-7 and ZR-75-1 cancer cells. The results of tertiary mammospheres development experiment showed that there was a significant increase in the tumorsphere formation capacity was induced by oestrogen treatment, and this increase was abolished by ERα knockdown (Figure 3B). However, when YB-1 expression was rescued, the tumorsphere formation capacity was restored in two normal cancer cell lines (Figure 3B). We next examined the influence of the ERα/YB-1 axis on the tumorigenic capacity of MCF-7 CSCs. Treatment with oestrogen could significantly increase the tumorigenic capacity of MCF-7 CSCs and promote the expression level of YB-1 in tumors (Figure 3C and D). However, when YB-1 was knocked down, the tumorigenesis of cells remained at a low level after oestrogen treatment (Figure 3C and D). We also examined the level of YB-1 in tumors, the immunohistochemical results indicate that there was a distinct increase level of YB-1 in oestrogen treated tumor (Figure 3E). These results indicate that the activation of estrogen signaling can upregulate the stemness of ER-positive CSCs by promoting the expression of YB-1.

### Both the ERα and YB-1 proteins were downregulated during the differentiation of ER-positive CSCs

**Figure 4:**
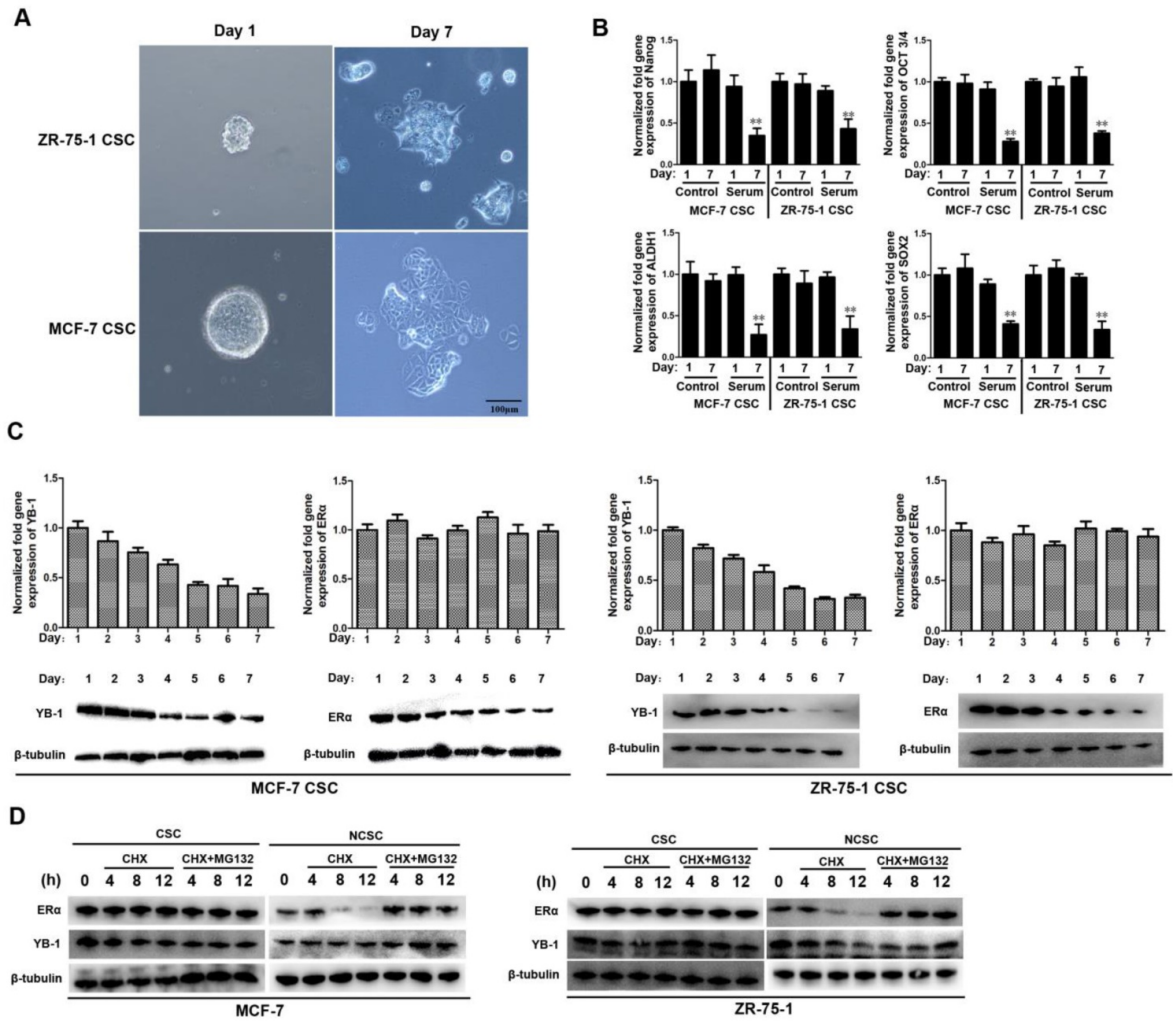
** Both the ERα and YB-1 proteins were downregulated during the differentiation of ER-positive CSCs.** (A) Induced differentiation of ER-positive breast CSCs. After exposure to 1% FBS for 7 days, cells were examined under a light microscope. Scale bar, 100 μm. (B) The expression level of stemness-related genes during cell differentiation. After exposure to 1% FBS for 7 days, the expression levels of stemness-related genes in cells were measured by quantitative real-time PCR (**, *p*<0.01). (C) The expression levels of YB-1 and ERα during cell differentiation. The expression levels of YB-1 and ERα in cells were measured by quantitative real-time PCR (top) and western blotting (bottom). β-Tubulin was used as the control. (**, *p*<0.01). (D) Western blots showing ER and YB-1 protein stability in the presence of cycloheximide (CHX) with or without MG-132 in ER-positive breast CSCs and NCSCs. β-Tubulin was used as the control.

To evaluate the protein levels of ERα and YB-1 during the differentiation of ER-positive CSCs, 1% FBS was added to the CSC culture medium to induce cell differentiation. The tumorsphere formation assay results indicated that the sphere-forming capacities of MCF-7 and ZR-75-1 CSCs were significantly suppressed when 1% FBS was added for 7 days (Figure 4A). The quantitative real-time PCR data revealed that the mRNA levels of stemness-related genes were significantly decreased in the FBS-treated group compared with the control group on day 7 (Figure 4B). Our findings indicate that ER-positive CSCs began to differentiate when exposed to 1% FBS for 7 days.

We further evaluated the mRNA and protein levels of ERα and YB-1 during the differentiation of MCF-7 and ZR-75-1 CSCs. The western blot analysis and quantitative real-time PCR results indicated that both the mRNA and protein levels of YB-1 gradually decreased during cell differentiation (Figure 4C). In contrast, during the process of cell differentiation, the protein level of ERα gradually decreased, but the mRNA level did not change significantly (Figure 4C). We sought to determine whether the protein level of ERα was influenced by protein degradation in differentiated cells. Indeed, the half-life of ERα in CSCs was significantly longer than that in NCSCs (Figure 4D). In contrast, there was no significant difference in the half-life of YB-1 between CSCs and NCSCs (Figure 4D). Treatment with the proteasome inhibitor MG-132 increased the half-life of ERα in NCSCs (Figure 4D). Therefore, these findings indicate the existence of a special proteasome degradation pathway for the ER protein in differentiated cells (NCSCs).

### YB-1 promotes proteasomal ERα degradation in differentiated cells

**Figure 5:**
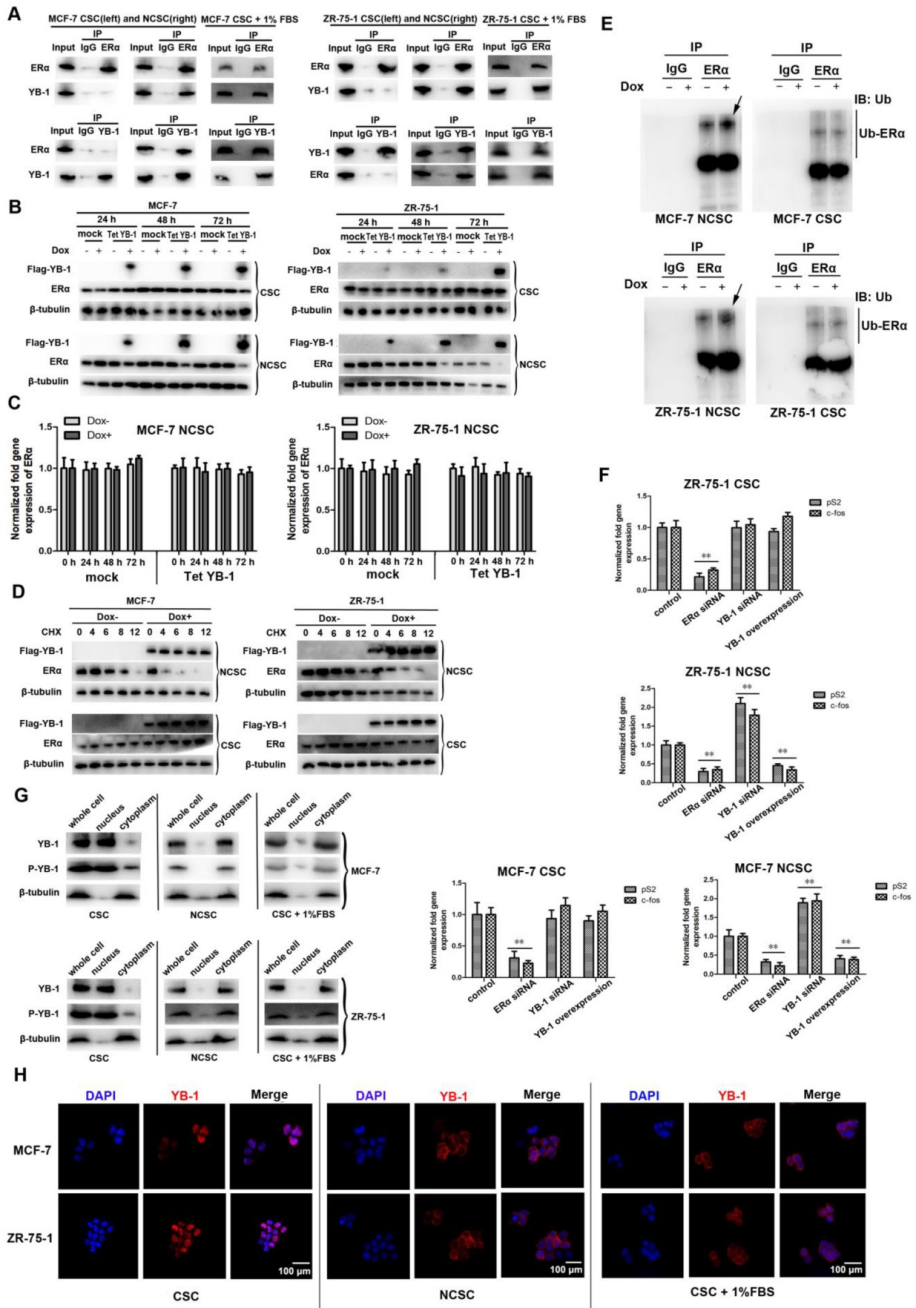
** YB-1 promotes proteasomal ERα degradation in differentiated cells.** (A) Co-IP assays showing YB-1 binding to ERα in ER-positive breast NCSCs. (B) Expression levels of YB-1 and ERα after YB-1 induction via the YBX1/Tet-On expression vector in ER-positive breast CSCs and NCSCs. After YB-1 was induced by doxycycline (Dox) for 24, 48 and 72 h, protein expression was assessed by western blotting. β-Tubulin was used as the control. (C) Impact of YB-1 induction on the mRNA level of ERα in ER-positive breast NCSCs. Seventy-two hours after YB-1 induction, the mRNA level of ERα in cells was measured by quantitative real-time PCR (**, *p*<0.01). (D) ERα protein stability in the presence of CHX after treatment with or without Dox. Protein expression was assessed by western blotting. β-Tubulin was used as the control. (E) ER ubiquitination with MG-132 after treatment with or without Dox for 48 h in ER-positive breast CSCs and NCSCs. Co-IP using an anti-ERα antibody and immunoblotting (IB) with an anti-ubiquitin (Ub) antibody. The arrows indicate increased levels of ubiquitination in NCSCs after Dox treatment. (F) Impact of YB-1 on the expression of ERα target genes in ER-positive breast CSCs and NCSCs. Seventy-two hours after YB-1 siRNA/ERα siRNA/pcDNA-YB-1 plasmid transfection, the mRNA levels of ERα target genes in cells were measured by quantitative real-time PCR (**, *p*<0.01). (G) The phosphorylation level and karyoplasmic distribution of YB-1 in CSCs and differentiated cells.β-Tubulin was used as the control. (H) The karyoplasmic distribution of YB-1 in CSCs and differentiated cells by immunofluorescence analyze. The YB-1 protein was detected with a YB-1-specific fluorescent antibody (red). Nuclei were stained with DAPI (blue).

YB-1 has previously been reported to negatively regulate ERα 38, 39. Here, coimmunoprecipitation experiments were performed to determine whether YB-1 could be confirmed as an ERα binding partner in differentiated cells by western blotting, revealing that YB-1 bound ERα in differentiated cells (NCSCs). In contrast, there was no significant direct interaction between YB-1 and ERα in MCF-7 and ZR-75-1 CSCs (Figure 5A). More importantly, 1% FBS induced YB-1 to interact with ERα in breast CSCs (Figure 5A). We sought to determine whether the degradation of ERα in NCSCs was influenced by YB-1. YB-1 was highly expressed in YB-1/Tet-On-transfected cells compared with control cells (Figure 5B). ERα levels decreased after the induction of YB-1 expression in NCSCs (Figure 5B), whereas the ERα mRNA levels remained unchanged (Figure 5C). In contrast, the expression of YB-1 had no effect on the protein levels of ERα in MCF-7 and ZR-75-1 CSCs (Figure 5B). We also examined the change in the half-life of ERα with or without YB-1 induction, revealing that the half-life of ERα in NCSCs was significantly decreased when YB-1 expression was induced (Figure 5D). Furthermore, ubiquitination of ERα increased when YB-1 expression was induced in NCSCs (Figure 5E). In contrast, ubiquitination of ERα was unchanged in stem cells (Figure 5E). We investigated whether YB-1 can influence the target genes of ERα in NCSCs. When YB-1 was transiently overexpressed in MCF-7 and ZR-75-1 NCSCs, the levels of the ERα target genes pS2 and c-fos were significantly reduced compared with those in control cells (Figure 5F) 39, 40. Conversely, YB-1 overexpression in CSCs did not influence the expression levels of pS2 and c-fos (Figure 5F). We further analyzed the phosphorylation level and karyoplasmic distribution of YB-1 in CSCs and differentiated cells by western blot, revealing that the cell differentiation induced by FBS could inhibit YB-1 phosphorylation and promote YB-1 protein transfer from the nucleus to the cytoplasm (Figure 5G). The karyoplasmic distribution of YB-1 was also confirmed by immunofluorescence experiments (Figure 5H). Taken together, these findings indicated that YB-1 can induce ERα degradation by ubiquitination via directly interacting with ERα in differentiated cells.

### The influence of YB-1 on the drug sensitivity of ER-positive CSCs

**Figure 6:**
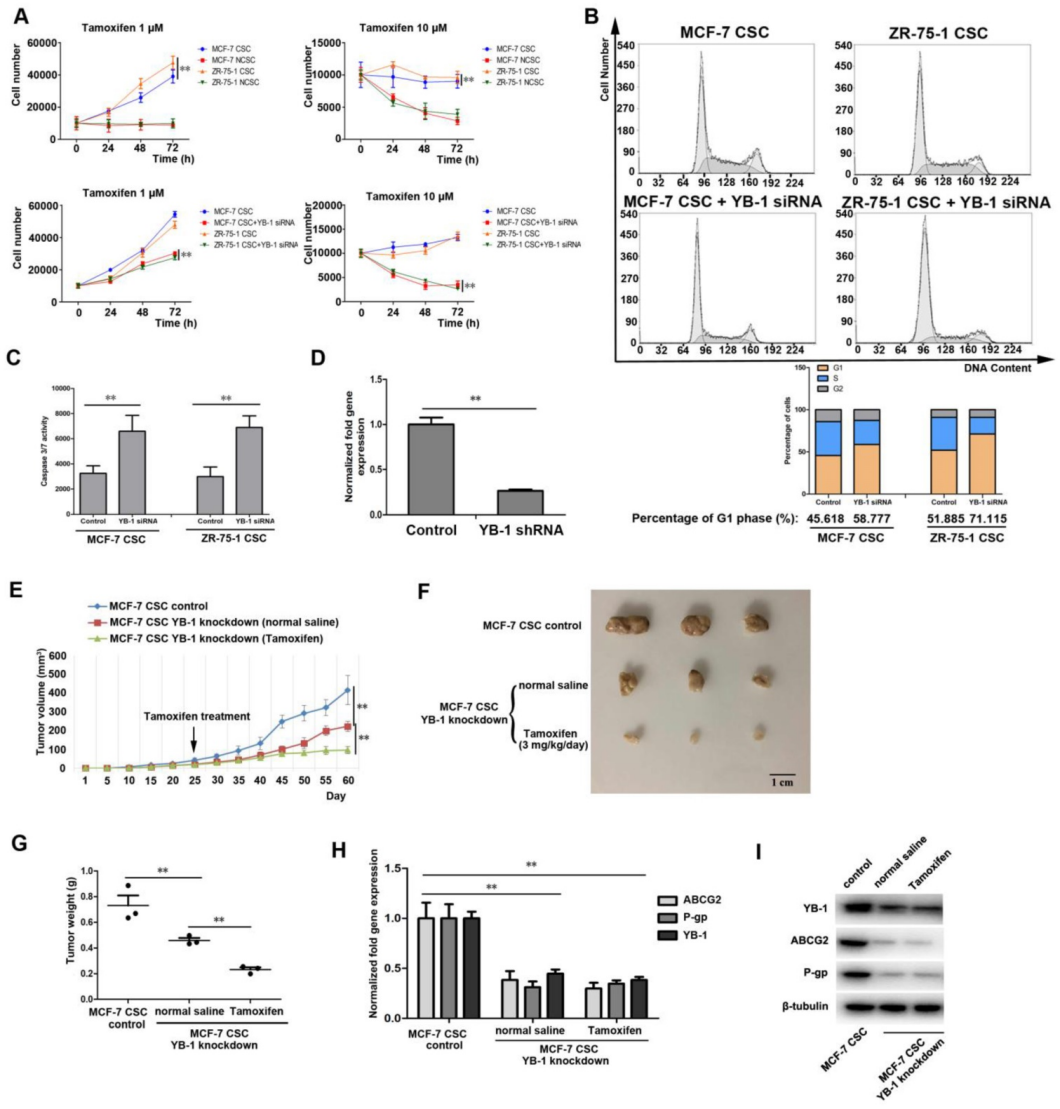
** The influence of YB-1 on the drug sensitivity of ER-positive CSCs.** (A) Impact of YB-1 on proliferation activity in ER-positive breast CSCs treated with 1 or 10 μM tamoxifen. Cells were seeded in a 6-well plate at 1×10^4^ cells/well. At different times after transfection with YB-1 siRNA, the cells were counted. The experiments were carried out in triplicate (**, *p*<0.01). (B) The influence of YB-1 on the cell cycle of ER-positive breast CSCs treated with 10 μM tamoxifen. After transfection with YB-1 siRNA for 36 h, the percentage of cells (1×10^4^) in the G1 phase was determined by flow cytometry. (C) The influence of YB-1 on caspase 3/7 activity in ER-positive breast CSCs treated with 10 μM tamoxifen. Caspase 3/7 activity in cells was detected after transfection with YB-1 siRNA for 36 h (**, *p*<0.01). (D) The expression level of YB-1 in MCF-7 CSCs after treatment with YB-1 shRNA. The mRNA level of YB-1 in MCF-7 CSCs was measured by quantitative real-time PCR (**, *p*<0.01). (E) Effects of YB-1 knockdown on tumor growth in mice with or without tamoxifen treatment. The mice were administered tamoxifen (3 mg/kg/day) via gavage on day 25. The tumor volumes in the mice were measured every 5 days. Sixty days later, the mice were sacrificed. The mean level in 3 mice is indicated (**, *p*<0.01). (F) Influence of YB-1 knockdown on solid tumors in mice with or without tamoxifen treatment. A solid tumor was collected from each mouse. (G) Impact of YB-1 knockdown on tumor weight in mice with or without tamoxifen treatment. The data are presented as the mean tumor weights for 3 mice (**, *p*<0.01). (H) Influence of YB-1 knockdown on multidrug resistance-related genes in solid tumors. The mRNA levels of multidrug resistance-related genes in solid tumors were measured by quantitative real-time PCR (**, *p*<0.01). (I) Influence of YB-1 knockdown on multidrug resistance genes in solid tumors. The protein levels of multidrug resistance-related genes in solid tumors were measured by western blotting. β-Tubulin was used as the control.

The above evidence suggests that YB-1 is a key factor that maintains the stemness of ER-positive CSCs, and CSCs with stemness features are thought to be responsible for resistance to chemotherapy 41. Indeed, the results of the cell proliferation assay indicated that the proliferation capacity of MCF-7 and ZR-75-1 CSCs was significantly higher than that of NCSCs treated with 1 or 10 μM TAM (Figure 6A), indicating that ER-positive CSCs are more resistant to TAM than NCSCs. To evaluate the effect of YB-1 on the TAM resistance of ER-positive CSCs, YB-1 expression was knocked down, and cell proliferation was then evaluated. The results showed that the low YB-1 level in stem cells treated with YB-1-siRNA significantly reduced the proliferation rate of MCF-7 and ZR-75-1 CSCs treated with 1 or 10 μM TAM (Figure 6A). We sought to determine whether knockdown of YB-1 in CSCs could influence the cell cycle in the presence of TAM, and we characterized the cell cycle of MCF-7 and ZR-75-1 CSCs by flow cytometry. The results showed that the percentage of YB-1 knockdown cells in the G1 phase was significantly increased compared with that of control cells (Figure 6B). More importantly, the level of caspase 3/7 activity, a known indicator of apoptosis, was increased in YB-1 knockdown CSCs treated with TAM (Figure 6C).

We then examined whether YB-1 knockdown could inhibit the tumorigenesis of ER-positive CSCs treated with TAM. To this end, YB-1 expression was knocked down, and cells were injected into nude mice. Quantitative real-time PCR showed that the YB-1 mRNA level was very low in MCF-7 CSCs transfected with YB-1-specific shRNA (YB-1-shRNA) (Figure 6D). Our results indicated that the volume, size and weight of tumors formed by YB-1 knockdown cells were significantly reduced in mice treated with TAM compared with those in mice treated with saline solution, while MCF-7 CSCs generated the largest tumors in mice (Figure 6E, F and G). These results indicated that YB-1 knockdown not only inhibited the tumorigenic ability but also increased the TAM sensitivity of MCF-7 CSCs. Next, we examined the expression level of YB-1 target genes associated with drug resistance (ABCG2 and P-gp) in tumors 24, 25. The quantitative real-time PCR and western blotting results showed that ABCG2 and P-gp expression was significantly reduced by YB-1 knockdown compared with that in control cells (Figure 6H and I).

Taken together, these findings indicated that YB-1 knockdown increases the sensitivity of ER-positive CSCs to TAM in vitro and in vivo.

## Discussion

YB-1 is a well-known oncogenic transcription factor involved in the regulation of cell proliferation, malignant transformation, cell metastasis and multidrug resistance 24-30. More recently, growing evidence has indicated that the enhancement of cancer stem cell characteristics has a close relationship with YB-1 expression in cancer cells. For example, a previous study showed that blocking YB-1 protein expression delayed tumorigenesis in mice and that YB-1 was involved in the expression of stemness surface markers, including CD44, in breast cancer-initiating cells 42. Wu et al. reported that the cell fate determination factor Dachshund (DACH1) suppressed epithelial-to-mesenchymal transition (EMT) and cancer stem cell-like characteristics by inactivating YB-1, suggesting that YB-1 is an important stemness factor in breast cancer cells 43. Our previous studies also found that YB-1 was significantly highly expressed in melanoma and breast CSCs and involved in the activation of the Notch and WNT signaling pathways 31, 32. Although the importance of YB-1 in maintaining stemness in CSCs is well documented, the regulatory mechanism of YB-1 expression in CSCs has, until recently, not been investigated. Herein, our results revealed that YB-1 was highly expressed and had major effects on stemness in ER-positive breast CSCs. In short, the decision of CSCs to undergo either self-renewal or differentiation is tightly controlled by the YB-1 level. High YB-1 levels promote self-renewal and stemness, whereas low YB-1 levels inhibit stemness and promote cell differentiation. To explore the mechanism underlying the high expression of YB-1 in ER-positive breast CSCs, we predicted and identified a transcription factor, ERα, that regulates the expression of YB-1 in ER-positive breast CSCs. Our study showed that activated ERα protein facilitates YB-1 expression and cell stemness by directly binding to the YB-1 promoter. Our current results are consistent with those of another report about the relationship between ERα signaling and stemness maintenance in breast cancer.

Breast CSCs are thought to be responsible for breast tumor growth 44. These CSCs can drive tumor growth and possess multilineage differentiation potential. Similar to normal stem cells, in tumors, CSCs can undergo both self-renewing symmetric division (producing two CSC daughter cells) and differentiating asymmetric division (producing a CSC daughter cell and a differentiated NCSC daughter cell) 44, 45. The regulatory mechanism by which CSCs undergo either symmetric or asymmetric division is not clear. The findings of this study highlight YB-1 as the core factor that regulates stemness in ER-positive CSCs. We further explored how breast cancer stem cells regulate cell differentiation by downregulating the expression of YB-1, revealing that the protein levels of ERα and YB-1 protein in ER-positive breast CSCs were stable. By inducing cell differentiation, we found that the ERα protein exhibits a specific proteasomal degradation pathway in highly differentiated ER-positive breast cancer cells. Furthermore, the mRNA and protein levels of YB-1 were gradually downregulated during cell differentiation. Since we found that ERα is a transcription factor regulating YB-1, the downregulation of YB-1 expression during cell differentiation is obviously related to the protein degradation of ERα. Interestingly, some previous studies have reported that YB-1 is a negative regulator of ERα 38, 39, and a negative feedback mechanism appears to exist between the two proteins. Indeed, we observed that YB-1 directly interacts with ERα and promotes the degradation of ERα by ubiquitination specifically in differentiated cells. In contrast, there was no significant direct interaction between YB-1 and ERα in CSCs. Thus, we believe that the initiation of ER-positive breast CSC differentiation is related to the interaction of the YB-1 and ERα proteins. When YB-1 interacts with ERα, proteasomal degradation of the ERα protein begins, leading to a sustained decline in the ERα protein level and further inhibition of the expression of YB-1 protein. Therefore, the cells maintain YB-1 protein expression at a relatively low level by regulating the interaction between the YB-1 and ER proteins, thus promoting cell differentiation.

An important unanswered question is what causes YB-1 to bind to the ERα protein when the cell initiates the differentiation process. It is well known that the cellular localization of the YB-1 protein is closely related to stemness regulation 43. As a transcription factor, YB-1 protein localization in the nucleus enforces CSC-like properties through transcriptional upregulation of NANOG, a marker of CSCs required for invasion and sphere formation 46. YB-1 also regulates SOX2 in breast CSCs, maintaining stem-like properties and tumorigenic potential 47. It is worth noting that YB-1 nuclear translocation could be facilitated by its phosphorylation. For example, there is evidence showing that YB-1 is phosphorylated at Ser102 by the serine/threonine kinase AKT before being shuttled to the nucleus 48. Supporting this, inhibition of p90 RSK, a major kinase involved in YB-1 phosphorylation, using the small molecule LJI308 eradicated the population of breast CSCs and induced the apoptosis of breast cancer cells 49. Therefore, we hypothesized that the dephosphorylation and cytoplasmic localization of YB-1 might be associated with cell differentiation. Herein, analysis of the phosphorylation level of YB-1 during cell differentiation revealed that serum not only inhibited the phosphorylation level of YB-1 in CSCs and facilitated its transfer to the cytoplasm but also promoted the interaction between the YB-1 and ERα proteins. Therefore, our study suggests that ER-positive breast CSCs initiate cell differentiation by regulating the dephosphorylation of YB-1. Specifically, the downregulation of YB-1 phosphorylation leads to its transfer from the nucleus to the cytoplasm and binding to the ERα protein, which in turn promotes ubiquitination and degradation of the ERα protein. Since ERα is a transcription factor that regulates the expression of YB-1, the degradation of ERα directly leads to the reduction in YB-1 protein levels. Due to the dual decrease in YB-1 phosphorylation and total protein levels, cells gradually lose their stemness characteristics and move toward cell differentiation. Therefore, the dephosphorylation of YB-1 and the interaction between YB-1 and ERα may be the switch that initiates the differentiation of ER-positive CSCs.

The ligand-activated transcription factor ERα is a key driver of the breast cancer phenotype in 60~70% of patients 2. Thus, blocking ER function is the main targeted therapeutic strategy employed in ER-positive breast cancer 3-5. The selective ER modulator TAM has been the mainstay of endocrine therapy in breast cancer patients for almost three decades 5. Unfortunately, many patients who receive TAM as adjuvant therapy eventually acquire TAM resistance 3. Studies have shown that one of the causes of increased TAM resistance may be the presence of CSCs 7. Indeed, our results showed that ER-positive CSCs were more resistant to TAM than NCSCs. We believe that the induction of ER-positive CSC differentiation is an effective method for improving the sensitivity of cells to TAM. In this study, we revealed a role for YB-1 in TAM resistance in ER-positive CSCs. Differentiation of ER-positive CSCs induced by YB-1 knockdown significantly increased the sensitivity of cells to TAM in vitro and in vivo. Furthermore, knockdown of YB-1 significantly decreased the levels of the drug resistance target genes ABCG2 and P-gp. It is now generally recognized that P-gp and ABCG2 play important protective roles in CSCs against exposure to drugs and xenobiotics. Collectively, cell differentiation and decreased multidrug resistance may be the important factors underlying the sensitivity of CSCs to TAM. Therefore, YB-1 may be an effective target for the treatment of ER-positive breast CSCs.

## Supplementary Material

Supplementary figures and tables.

## Figures and Tables

**Figure 7:**
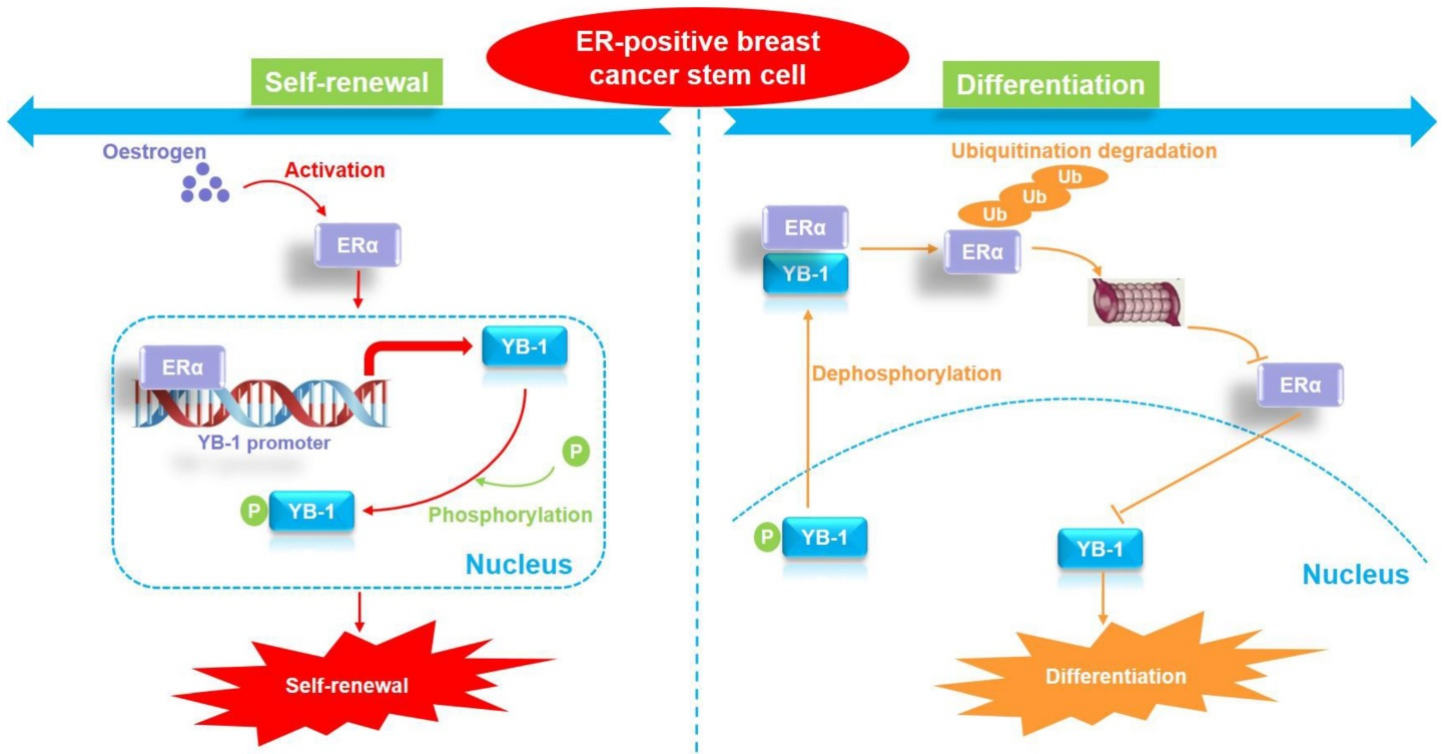
The proposed model of YB-1 interaction with ERα to regulate the stemness and differentiation of ER-positive breast cancer stem cells.
