# Supplementary material for: YB-1 interplays with ERα to regulate the stemness and differentiation of ER-positive breast cancer stem cells
Source: Theranostics. 2020 Feb 19;10(8):3816–32. doi: 10.7150/thno.41014 (PMC7069074; doi:10.7150/thno.41014)

Supplementary Table 1

Table 1. The sequences of nucleotide and primers used in the study

Name	Sequence (5’→3’)
YB-1 siRNA	YB-1 siRNA-1: GGAACGGAUAUG GUUUCAUTT<br>YB-1 siRNA-2: CCAGTTCAAGGCAGTAAATAT
ERα siRNA	ERα siRNA: GGAGAAUGUUGAAACACAATT
YB-1 shRNA-1	Top strand:<br>GATCCGCCAGTTCAAGGCAGTAAATATTTCAAGAGAATA<br>TTTACTGCCTTGAACTGGTTTTTTG<br>Bottom strand:<br>AATTCAAAAAACCAGTTCAAGGCAGTAAATATTCTCTTG<br>AAATATTTACTGCCTTGAACTGGCG
YB-1 shRNA-2	Top strand:<br>GATCCGAGCAGACCGTAACCATTATAGTTCAAGAGACTA<br>TAATGGTTACGGTCTGCTTTTTTTG<br>Bottom strand:<br>AATTCAAAAAAAGCAGACCGTAACCATTATAGTCTCTTG<br>AACTATAATGGTTACGGTCTGCTCG
HAPDH	F: GGTATCGTGGAAGGACTCATGAC<br>R: ATGCCAGTGAGCTTCCCGTTCAG
ALDH1	F: TTACCTGTCCTACTCACCGA<br>R: CTCCTTATCTCCT TCTTCTACCT
ABCG2	F: GGCCTCAGGAAGACTTATGT<br>R: AAGGA GGTGGTGTAGCTGAT
OCT 3/4	F: GAGCAAAACCCGGAGGAGT<br>R: T TCTCTTTCGGGCCTGCAC
Nanog	F: GCTTGCCTTGCTTTGAAGCA<br>R: TTCTTGACTGGGACCTTGTC
CDH1	F: CAAATCCAACAAAGACAAAGAAGGC

	R: ACACAGCGTGAGAGAAGAGAGT
DSP	F: GTTTTGGGGCAGGTCAGGATT<br>R: GGGAGGATAAGCACCGAAGAA
ZO-1	F: AGCCATTCCCGAAGGAGTTGAG<br>R: ATCACAGTGTGGTAAGCGCAGC
Sox2	F: AAAATCCCATCACCCACAGCAA<br>R: AAA ATAGTCCCCCAAAAAGAAGTCC
E-cadherin	F: TGAAGGTGACAGAGCCTCTGG<br>R: TGGGTGAATTCGGGCTTGTT
DPA	F: ACGCCAGGATGATGACTGGA<br>R: GTCACTGCGAGCTTCTTTACAT
Cyclin A	F: CGCTGGCGGTACTGAAGTC<br>R: GAGGAACGGTGACATGCTCAT
YB-1	F: AGGCAGGAACGGTTGTAGGT<br>R: CCTTGTTCTCCTGCACCCTG
ERα	F: GGGAAGTATGGCTATGGAATCTG<br>R: TGGCTGGACACATATAGTCGTT
pS2	F: CCCCGTGAAAGACAGAATTGT<br>R: GGTGTCGTCGAAACAGCAG
c-fos	F: CACTCCAAGCGGAGACAGAC<br>R: AGGTCATCAGGGATCTTGCAG
P-gp	F: TTGCTGCTTACATTCAGGTTTCA<br>R: AGCCTATCTCCTGTCGCATTA
YB-1 promoter	F: AACGGAGTGTAGTCGGCTGACCCCT<br>R: ACTCTAAGACTTCTCTTAGAAGTCA
YB-1 protein-coding gene	F: ATGAGCAGCGAGGCCGAGACCCAGC<br>R: TTACTCAGCCCCGCCCTGCTCAGCC
ERα protein-coding gene	F: ATGACCATGACCCTCCACACCAAAG<br>R: TCAGACCGTGGCAGGGAAACCCTCT

Supplementary figure 1

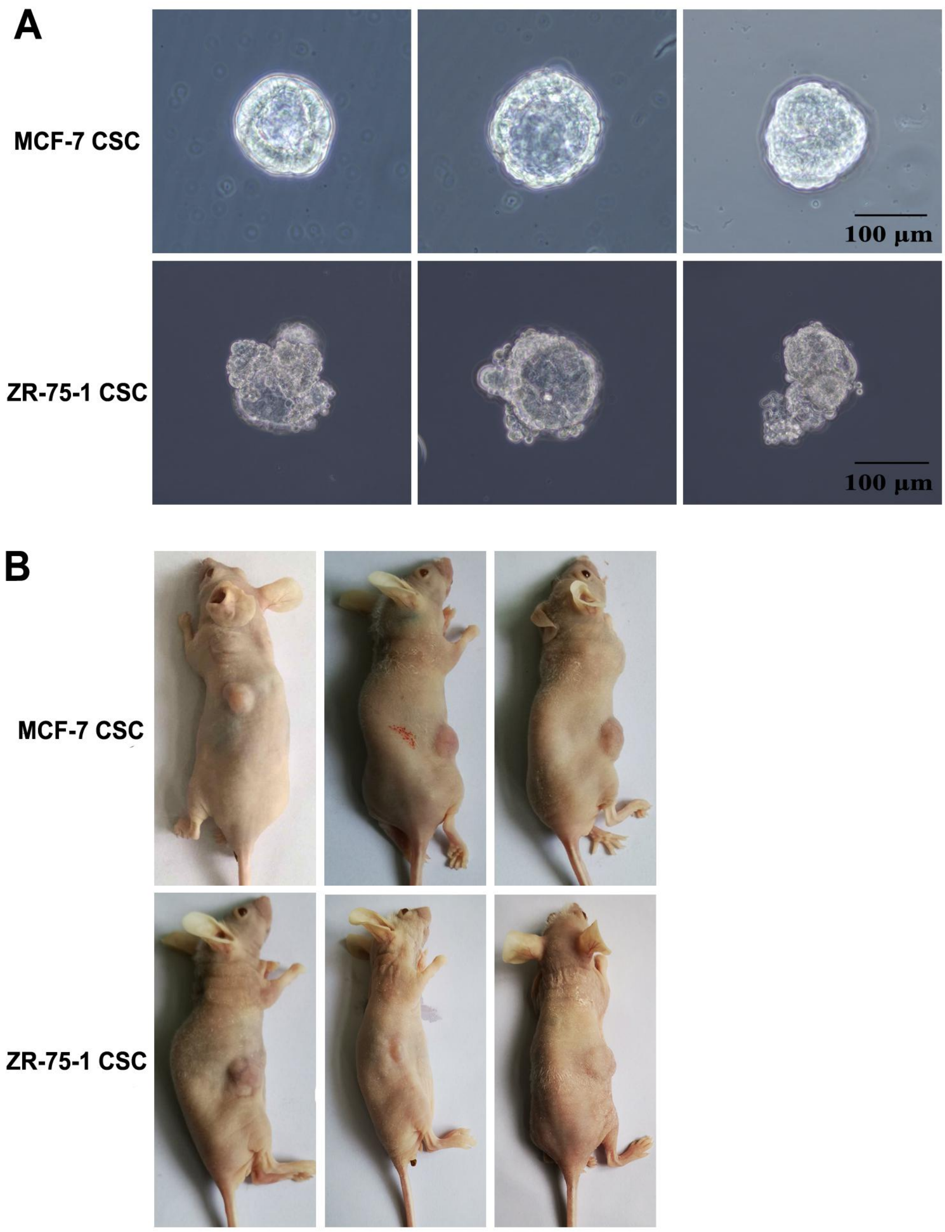

Supplement: Supplementary file 1 — Supplementary figures and tables. [file thnov10p3816s1.pdf]
